# Biological vs. Chronological Overnight Fasting: Influence of Last Evening Meal on Morning Glucose in Dysglycemia

**DOI:** 10.3390/nu17122026

**Published:** 2025-06-18

**Authors:** Diana A. Diaz-Rizzolo, Haley Yao, Leinys S. Santos-Báez, Collin J. Popp, Rabiah Borhan, Ana Sordi-Guth, Danny DeBonis, Emily N. C. Manoogian, Satchidananda Panda, Bin Cheng, Blandine Laferrère

**Affiliations:** 1 Division of Endocrinology, Diabetes Research Center, Columbia University Irving Medical Center, New York, NY 10032, USA; hjy2113@cumc.columbia.edu (H.Y.); lss2181@cumc.columbia.edu (L.S.S.-B.); rb3692@cumc.columbia.edu (R.B.); aps2244@cumc.columbia.edu (A.S.-G.); dgd2119@cumc.columbia.edu (D.D.); 2 Health Science Faculty, Universitat Oberta de Catalunya (UOC), 08018 Barcelona, Spain; 3 Department of Population Health, NYU Langone Health Grossman School of Medicine, New York, NY 10016, USA; collin.popp@nyulangone.org; 4 Regulatory Biology Laboratory, Salk Institute for Biological Studies, La Jolla, CA 92037, USA; emanoogian@salk.edu (E.N.C.M.); panda@salk.edu (S.P.); 5 Department of Biostatistics, Mailman School of Public Health, Columbia University, New York, NY 10032, USA; bc2159@cumc.columbia.edu

**Keywords:** last eating occasion, evening meal, carbohydrate, postprandial glucose response, fasting glucose, overnight glucose, insulin sensitivity, insulin resistance, prediabetes, type 2 diabetes

## Abstract

*Background/Objectives:* Nocturnal glucose regulation is a critical but underexplored determinant of next-day fasting glucose (FG), particularly in individuals with dysglycemia. This study examined the role of glucose levels after the last eating occasion (LEO) and during the overnight fast in predicting FG, considering the potential influence of carbohydrate content in LEO and insulin sensitivity. *Methods:* In a controlled 24 h protocol, 33 adults (50–75 years) with prediabetes or diet-controlled type 2 diabetes followed a standardized feeding schedule with meals at fixed times, including a LEO at 10:00 p.m. Continuous glucose monitoring was used to assess glucose during the 3 h postprandial period (LEO-PPGR) and two fasting intervals: chronological overnight fast (COF) and biological overnight fast (BOF). Associations with FG were tested using general linear models, adjusting for carbohydrate intake and insulin sensitivity (Matsuda index). *Results:* Glucose responses during LEO-PPGR—assessed by mean glucose, peak, and AUC—were strongly correlated with FG the next morning (r = 0.704, 0.535, and 0.708, *p* < 0.001). Similarly, glucose levels during COF and BOF were also correlated with FG (r = 0.878, *p* < 0.001 for both), but these associations weakened after adjustment for LEO carbohydrate content. The Matsuda index was positively correlated with glucose in all three periods (*p* < 0.05), yet its inclusion in the model attenuated all previously significant associations with FG. *Conclusions:* These findings suggest that the glycemic response to the last meal and subsequent overnight glucose levels contribute to next-day FG, but their impact is modulated by carbohydrate content and individual insulin sensitivity. Understanding nocturnal glycemic dynamics may inform strategies for improving metabolic outcomes in dysglycemia.

## 1. Introduction

Glucose homeostasis is essential for maintaining metabolic health. Abnormal blood glucose concentrations, especially in individuals with diabetes, are linked with serious health risks outcomes like cardiovascular disease, kidney failure, neuropathy, retinopathy, and higher mortality. Persistent hyperglycemia induces a rapid vascular damage and inflammation, and may lead to more complications. Thus, understanding factors that influence glycemia is important to develop preventing measures to decrease risk. Fasting glucose (FG) levels are significant predictors of disease progression in prediabetes and type 2 diabetes, and one of the targets of diabetes management [1]. Meal carbohydrate content is one of the main determinants of postprandial glucose response (PPGR) [2]. The carbohydrate content of the last eating occasion of the day (LEO), and the subsequent LEO-PPGR, have been shown to significantly contribute to next-day FG levels in patients with type 2 diabetes and elevated HbA1c (>9%) [3].

Overnight glucose levels reflect the body’s ability to maintain glucose homeostasis during fasting periods, which is critical for overall metabolic health [4]. During the overnight fasting state, metabolic processes, such as hepatic gluconeogenesis and glycogenolysis, are essential for preventing hypo- or hyperglycemia [5]. Nocturnal glucose is not only a passive reflection of the carbohydrate content consumed during the LEO [6]; it also integrates the body’s broader metabolic control mechanisms [7].

The significance of overnight glucose control extends beyond its immediate impact on next-day FG: stable overnight glucose levels are linked to better overall glucose control and a reduced risk of long-term complications associated with diabetes [8,9]. In recent years, continuous glucose monitoring (CGM) has become an important tool in the management of diabetes, passively providing real-time information on glucose fluctuations. CGMs provide feedback on detailed glucose patterns in people with prediabetes and type 2 diabetes, enabling healthcare providers to implement precision nutrition interventions tailored to specific patient needs [10]. Elucidating the determinants of glucose variability, especially during nocturnal periods, will better inform these precision intervention strategies aimed at improved glycemic control [11]. However, the existing literature has primarily focused on the role of LEO’s carbohydrate content in modulating postprandial glucose levels in type 1 diabetes [12], with limited evidence in adults with type 2 diabetes [13]. Moreover, to our knowledge, the contribution of nocturnal fasted glucose levels, independent of LEO’s timing or macronutrient composition, to FG has not been studied extensively.

The duration of the overnight fast, i.e., of the daily eating window, has recently become an area of research focus. Eating for prolonged periods during the day, i.e., having a short overnight fast, has been associated with obesity and dysmetabolism [14]. It is of utmost importance to be able to measure the duration of the overnight fast as well as define a true fasting period. Here, we defined two specific fasting periods: the chronological overnight fast (COF), which refers to the time interval between the beginning of the LEO until wake up time the following morning, and the biological overnight fast (BOF), a stricter definition of the overnight fasting period that excludes glucose excursions after the last meal, and better reflects the body’s true fasting state. We hypothesize that the carbohydrate content of the LEO will positively influence the LEO-PPGR, and consequently, impact glucose levels during the COF, which in turn will be associated with next-day FG. However, glucose levels during the BOF will not be affected by LEO and will be a lesser determinant of next-day FG.

By providing a more granular assessment of glucose levels during overnight periods, we investigated how the timing and the carbohydrate content of the LEO influence nocturnal glucose dynamics and next day FG in individuals with dysglycemia. We aimed to clarify the relationship between LEO composition, overnight glucose regulation, and insulin sensitivity, providing new insights into factors affecting glucose homeostasis in this population and perhaps disease progression.

## 2. Materials and Methods

### 2.1. Study Design

Data were collected during a baseline assessment as part of the New York Time-Restricted EATing (NY-TREAT) parent trial (June 2021–February 2024) (Clinical Trials registration: NCT04465721) [15]. Participants, aged 50-75 years old, with obesity or overweight and diet and metformin-controlled prediabetes or type 2 diabetes with HbA1c of 5.7–7.5%, a ≥14 h daily eating window, with ≥6 h of sleep and no history of sleep disorders nor shift work, with stable weight, and no history of bariatric surgery, enrolled in the parent NY-TREAT trial after signing an informed consent. Data from 33 participants enrolled in the NY-TREAT trial at the time of this exploratory investigation were used for this analysis. No formal sample size calculation was performed, as this study aimed to generate preliminary insights into the relationship between the last evening meal and overnight glucose metabolism. The findings provide an initial framework for hypothesis generation, which can inform future studies with larger sample sizes and power calculations. Participant consent was collected at Columbia University Irving Medical Center (New York City) and all procedures were approved by the Columbia University Institutional Review Board.

The controlled 24 h research diet was the only controlled intervention part of a 2-week baseline assessment period occurring under free-living conditions, i.e., under habitual eating, sleeping, and physical activity behavior, from day 1 to day 12. On the first day of the 2-week assessment period, participants were fitted with a blinded CGM (Abbott Freestyle Libre Pro, Abbott Park, IL, USA) to track their glucose levels, and an actigraph to monitor sleep (GT3X, ActiGraph LLC, Pensacola, FL, USA), validated with a sleep log. Neither bedtime nor wake-up time were controlled. Participants were instructed to log all meals in real-time using the myCircadianClock (mCC) app [16], by taking a time-stamped photograph at the start of each meal. On day 13, participants came to the Clinical Research Center (CRC) for a 24 h controlled-diet intervention, and returned the following morning on day 14, after a 10 h overnight fast, for a 2 h 75 g Oral Glucose Tolerance Test (OGTT). An intravenous catheter was inserted in an antecubital vein by a research nurse. Blood samples were obtained at −15 and 0 min immediately before a 75 g glucose drink at 8:00 a.m., and again at 15, 30, 60, 90, and 120 min after the drink, before being centrifuged, aliquoted, and stored at −80 °C.

### 2.2. 24-H Controlled Diet

On day 13, the diet-controlled day, participants consumed a time-, composition-, and calorie-controlled eucaloric diet. The diet was personalized based on each individual’s energy requirements calculated using the Mifflin-St. Jeor equation [17,18]. The overall macronutrient composition of the diet consisted of 56–59% carbohydrates, 14–17% protein, and 26–28% fat. The caloric distribution was allocated as 30% of daily calories for breakfast, lunch, and dinner, with the remaining 10% designated for the evening snack (LEO).

While the total daily caloric intake and macronutrient distribution were standardized, the distribution of macronutrients across meals varied according to the dietary condition assigned to each participant. As a result, participants received different food items and nutrient composition at LEO, adapted to their individual energy needs and diet condition. Therefore, the carbohydrate content of LEO varied among participants, ranging from 6 to 56 g, and from 14% to 88.5% of the total meal caloric balance. This variability allowed us to investigate the metabolic effects of different absolute and relative carbohydrate loads at the last meal, in relation to protein and fat intake. Meal timing was strictly regulated, with breakfast (9:00 a.m.) and lunch (1:00 p.m.) consumed in the CRC and dinner (6:00 p.m.) and LEO (10:00 p.m.) consumed at home. Participants were instructed to eat all food provided, without any instruction as to order of consumption of the various items in each meal, log all meals with the app, and return the containers the following day for inspection to ensure compliance.

### 2.3. Variable Determination and Measurement

**Figure 1:**
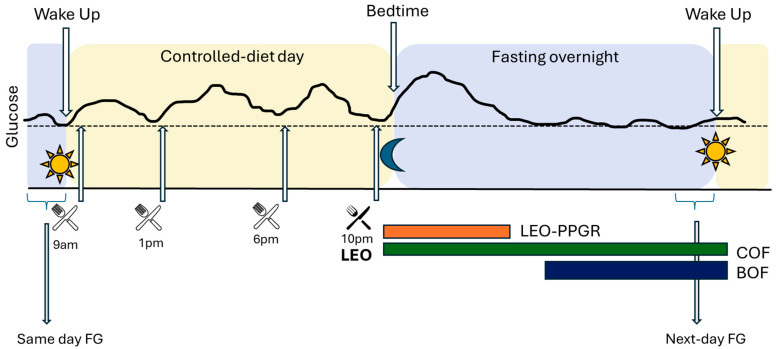
Study design and analyzed variables. BOF: biological overnight fast; COF: chronological overnight fast; FG: fasting glucose; LEO: last eating occasion; LEO-PPGR: fixed 3 h post prandial glucose response following meal. CGM: continuous glucose monitor glucose values: continuous black line.

The study design and the variables analyzed are depicted in Figure 1.

- Last Eating Occasion (LEO)—Postprandial Glucose Response (PPGR): a standardized fixed 3 h glucose excursion, beginning with the first CGM reading immediately after the initiation of LEO, and terminating 3 h after.
- Chronological Overnight Fast (COF): starting at the beginning of the LEO, captured by the mCC app, until wake time the next morning, as measured by actigraph.
- Biological Overnight Fast (BOF): time between the return of glucose levels to same-day FG (day 13) after LEO excursion, and the FG reading at wake-time (as measured by actigraph) the next day (day 14). In other words, the BOF period excludes the individualized LEO excursion, not the 3 h fixed LEO-PPGR itself.
- CGM glucose outcomes: peak glucose as the maximum glucose read and total area under the curve (tAUC) calculated using the trapezoidal method during 3 h fixed LEO-PPGR; average glucose from reads in the defined period (LEO-PPGR, COF, or BOF); next-day Fasting Glucose (FG) calculated from the average of the 3 CGM reads before wake time, determined by actigraphy, on day 14.
- The Matsuda index was calculated with the ratio of plasma glucose and insulin concentrations during the OGTT, on day 14 [19].

### 2.4. Statistical Analysis

Statistical analyses presented data as mean (SD). To examine the relationship between average glucose (LEO-PPGR, COF, and BOF), peak glucose, and tAUC during LEO-PPGR, or on/with next-day FG and LEO-carbohydrates (g and percentage), a two-tailed Pearson correlation coefficient was employed. To analyze the impact of LEO-PPGR (average, peak, and tAUC) and average glucose levels during COF and BOF on next-day fasting glucose (FG), a General Linear Model (GLM) was employed. The next-day FG was set as the dependent variable, with glucose variables, carbohydrate content from LEO, measured in grams and as a percentage, sugar (in grams and percentage) and dietary fibers (in grams), and Matsuda index as covariates. The main effects of the glucose variables and carbohydrate content or Matsuda index were evaluated separately, along with their interactions, to determine whether the impact of glucose variables on next-day FG was influenced by the nutritional composition of LEO or the insulin sensitivity. Statistical significance was set at *p* value < 0.05 and B coefficients were reported to quantify the strength and direction of the associations. Analyses used IBM SPSS Statistics software version 29.0.0.0.

## 3. Results

A total of 33 participants, aged 60.4 (6.9) years, comprising 24 women, three with type 2 diabetes and four on metformin (two of them with type 2 diabetes), were studied. The mean waist circumference was 105.5 (14.5) cm, body weight was 93 (22.3) kg, BMI was 33.5 (6.6) kg/m^2^, and HbA1c was 5.97 (0.23)%.

The calorie and nutrient composition of LEO varied among participants, with a wide range of carbohydrates either as grams or percentage of total calories (Supplementary Table S1). The next-day FG average was 90.52 (12.99) mg/dL. LEO carbohydrate content in grams did not show a positive correlation with next day FG (r = −0.422, *p* = 0.072).

The fixed 3 h mean glucose after LEO (LEO-PPGR) was 91.77 (12.09) mg/dL, with a mean glucose peak of 104.85 (13.85) mg/dL and a tAUC of 17,938 (2379) mg.min/dL. There was a significant positive correlation between mean glucose, peak glucose, and tAUC glucose during LEO-PPGR and next-day FG (Table 1). Surprisingly, neither the 3 h average, the peak, nor the tAUC glucose after LEO correlated significantly with the carbohydrate content of LEO, whether measured in grams or as a percentage of total LEO energy intake (Supplementary Table S2). When performing a GLM with next-day FG as the dependent variable and both LEO-PPGR and LEO carbohydrate content as covariates, a significant interaction considering carbohydrates in grams was observed between next-day FG and tAUC during LEO-PPGR (B < −0.001, *p* = 0.048) (Supplementary Table S2). This suggests that the grams of carbohydrate content for both LEO-PPGR and LEO contribute significantly in combination to next-day FG. However, given the very small B coefficient, the effect size is minimal, indicating that the LEO-PPGR impact on next-day FG is mostly independent of the carbohydrate content of LEO, without synergistic effect on next-day FG.

**Figure 2:**
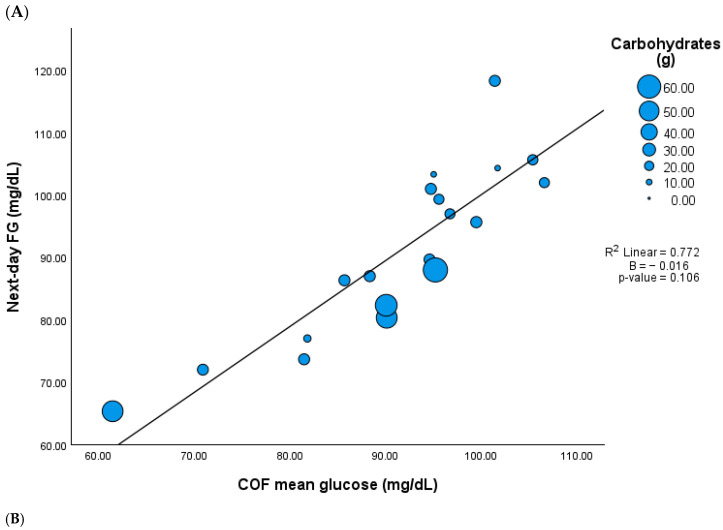

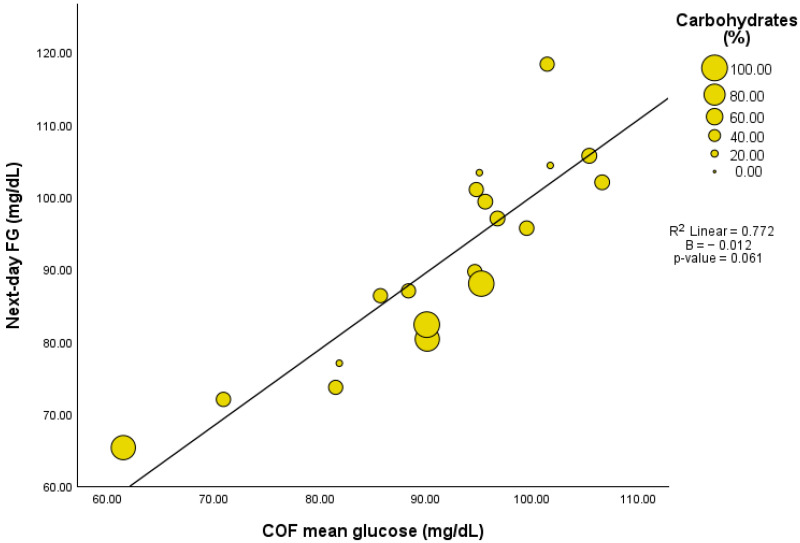
Association between mean glucose during chronological overnight fast (COF) and next day fasting glucose (FG) adjusted by LEO carbohydrate content (n = 19). (**A**) Carbohydrates in grams, (**B**) carbohydrates in percentage.

Out of 33 participants, 19 were eligible for the assessment of chronological and biological overnight fasting (COF and BOF) periods (Supplementary Table S3). The selected participants were eligible because they exhibited a glucose excursion in response to LEO that eventually returned to or fell below the defined day 13 FG levels, which is necessary to calculate COF and BOF. The mean COF period was 07 h:16 min (00 h:58 min) with a mean glucose level of 91.4 (11.4) mg/dL. While the mean glucose during COF did not correlate with neither the gram nor percentage of LEO’s carbohydrate content, it showed a positive correlation with next-day FG (r = 878, *p* < 0.001). Interestingly, the associations between COF and next-day FG disappear after adjustments for LEO’s carbohydrate content (Figure 2 and Supplementary Table S4).

The duration of the BOF period, which excludes the individualized glucose excursion following LEO, was 04 h:48 min (01 h:09 min), with a mean glucose level of 87.1 (12.5) mg/dL.

**Figure 3:**
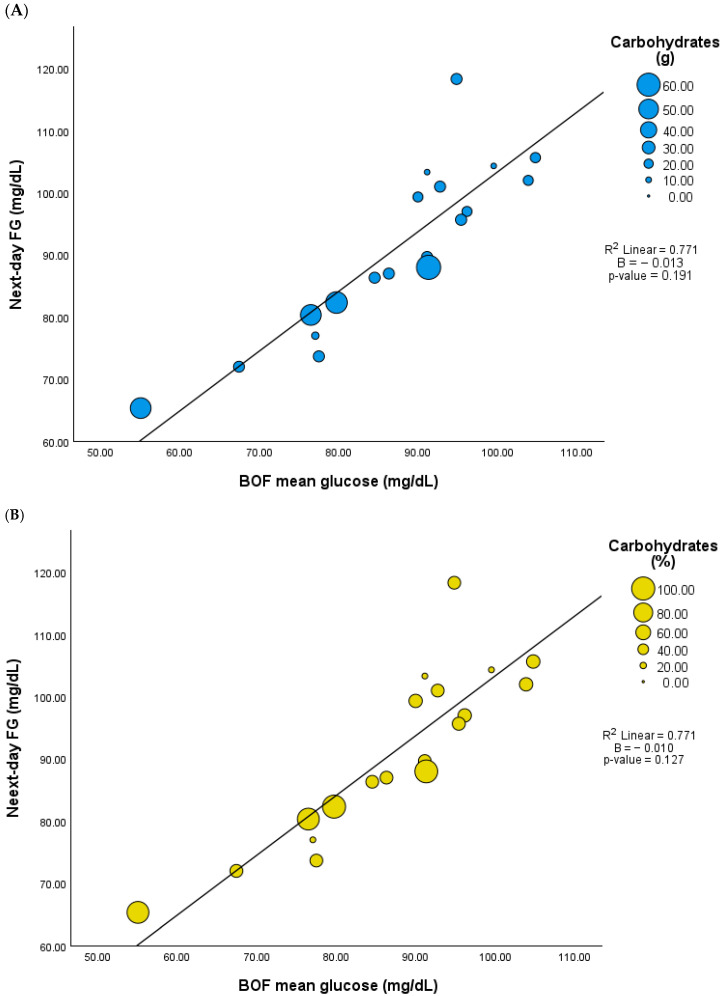
Association between mean glucose during biological overnight fast (BOF) and next-day fasting glucose (FG) adjusted by LEO carbohydrate content (n = 19). (**A**) Carbohydrates in grams, (**B**) carbohydrates in percentage.

Although BOF excludes the postprandial glucose response of LEO, mean glucose levels during BOF were positively correlated with LEO-PPGR, including 3 h fixed PPGR mean glucose (r = 0.815, *p* < 0.001), peak glucose (r = 0.505, *p* = 0.027), and tAUC glucose (r = 0.807, *p* < 0.001). Similarly to COF, the mean glucose during BOF did not correlate with LEO carbohydrate content either in grams or percentage (r = −0.437, *p* = 0.061 and r = −0.368, *p* = 0.120), but it did show a positive correlation with next-day FG (r = 0.878, *p* < 0.001). However, there was no significant association between BOF and next-day FG when the model included adjustments for the LEO carbohydrate content (Figure 3 and Supplementary Table S4).

All analyses were repeated with a more detailed breakdown of the carbohydrate content of LEO, with either dietary fiber in grams, or sugar content, both in grams and as a percentage of LEO total calories. Only the LEO dietary fiber content was significantly and positively correlated with next-day FG (r = 0.511, *p* = 0.001) and with LEO-PPGR (Supplementary Table S5). However, the associations between LEO-PPGR and next-day FG did not persist after adjusting for either LEO sugar or dietary fiber content. Similarly, associations between mean glucose during COF or BOF and next-day FG disappeared when adjusted for LEO sugar content or dietary fiber (Supplementary Table S5).

The Matsuda index was negatively correlated with 3 h fixed LEO-PPGR, either mean glucose (r = −0.423, *p* = 0.014), peak glucose (r = −0.419, *p* = 0.015), and tAUC glucose (r = −0.420, *p* = 0.015), and with mean glucose levels during COF (r = −0.559, *p* = 0.013) and BOF (r = −0.492, *p* = 0.032). The associations between glucose levels during LEO-PPGR, COF, and BOF with next-day FG did not persist after adjusting for the Matsuda index (Supplementary Table S6), indicating a role of insulin sensitivity in the association of nocturnal glucose and next day FG.

## 4. Discussion

Our study provides further evidence that fasting glucose regulation is not only influenced by the timing and nutrient composition of the evening meal, but also by nocturnal metabolic processes and insulin sensitivity in individuals with prediabetes and diet and/or metformin type 2 diabetes. Nocturnal fasting glucose showed a strong positive relationship with next morning glucose levels, indicating that the body’s ability to manage glucose overnight plays a significant role in determining glucose levels the next day. Glucose dysregulation during the nighttime has been observed even in healthy individuals [20]. This makes it especially relevant to study nocturnal glucose dynamics in populations with impaired glucose tolerance and diabetes.

In our study, the 24 h research diet was personalized to individual calorie requirements while maintaining a constant overall nutrient composition between participants. However, all meal times were standardized, fixed, and time-stamped with the app, including the LEO. This is important because meal timing has been shown to impact 24 h glucose levels [21]. Additionally, we controlled the percentage of calories distributed across the four meals on day 13, including LEO, as we had previously observed that specific calories distributions—predominantly in the evening—can influence glucose metabolism independently of fat mass, diet quantity, or quality [22]. This approach allowed us to isolate the effects of macronutrient composition, particularly carbohydrate content, on glucose regulation. Both PPGR and FG are interrelated [23]; PPGR levels have a substantial impact on HbA1c in well-controlled, non-insulin-treated type 2 diabetes patients [24], and a diet targeting the limitation of PPGR was shown to improve overall glycemic control in individuals with prediabetes [25].

Glucose tolerance is worse later in the day [26,27]. In our study, we observed that PPGR following the LEO was a predictor of next-morning FG. This initially supported the idea that the carbohydrate content of the last meal can lead to elevated postprandial glucose levels, subsequently influencing FG levels. It has been well established that meal carbohydrate content significantly affects PPGR [2]. We examined grams as well as the percentage of carbohydrates within the last meal; the latter is critical because post-meal glucose excursions can be attenuated with the addition of fat and protein to a meal [28]. This aligns with the growing evidence that meal composition, rather than carbohydrate content alone, is crucial for glucose management. Interestingly, we observed no relationship between the LEO’s carbohydrate and PPGR or glucose levels upon awakening the following morning. Previous studies have demonstrated that a carbohydrate-rich meal consumed as a bedtime snack can reduce morning hyperglycemia in non-insulin treated type 2 diabetes participants [29,30,31,32]. On the other hand, in an intervention study with 28 participants with type 2 diabetes, a high glycemic index during the evening meal was not related with higher PPGR and produced a lower overnight mean blood glucose concentration [13].

Additionally, carbohydrate consumption throughout the day has been shown to affect overnight glucose levels, influencing 24 h glucose profiles [33]. In our study, while the carbohydrate percentages remained stable throughout the day for all study participants, our results suggest the carbohydrate content of the LEO did not directly influence overnight glucose levels during chronological (COF) and biological (BOF) fasting periods. These findings indicate that nocturnal glucose regulation is influenced by factors beyond the nutritional composition of the last meal on the body’s ability to regulate nocturnal glucose.

Instead, both COF and BOF were strongly and positively associated with next-day FG, indicating that the body’s ability to manage glucose during overnight fasting plays a significant role in determining FG levels the following morning in individuals with diet and/or metformin-controlled prediabetes and type 2 diabetes. This is a novel finding, as previous research has primarily focused on carbohydrate content of the LEO as the key determinant of next-day FG. For instance, a previous study with eight healthy adults demonstrated that liver glycogen concentrations can significantly contribute to glucose production during fasting, indicating that glycogen stores are a primary source of glucose during the overnight fast. However, this study only tested participants following a low-carbohydrate diet, limiting its generalizability to different dietary patterns [34]. While we did not measure endogenous glucose production, our study is among the first to suggest the role of the control of glucose during a nocturnal fasting period (COF and BOF) on next-day FG. Concretely, previous studies have observed that a low-carbohydrate bedtime snack in type 2 diabetes individuals resulted in an increased hepatic insulin sensitivity, a decreased hepatic glucose production overnight, and lower fasting glucose the next morning [35]. Therefore, the persistence of the correlation between COF, and especially BOF, without the glucose excursion after LEO, and next-day FG, underscores the importance of metabolic processes during the deeper/pure fasting period, when the body relies on gluconeogenesis and glycogenolysis for endogenous glucose production [36]. However, the diminished positive relationship between COF and BOF with next-morning glucose levels when adjusting for the LEO carbohydrate content indicates that while carbohydrate intake during LEO may not directly influence next-day FG, it interacts with nocturnal glucose levels, which ultimately affect morning glucose levels.

Glucose homeostasis during the fasting state, as specifically represented by BOF, has been demonstrated to be a relevant predictor of next-day FG, independent of carbohydrate intake during LEO. This observation also reinforces that additional metabolic factors, such as insulin sensitivity, may exert a stronger influence during this fasting period due to the effectiveness of glucose uptake and utilization [37]. In this analysis, we observed that insulin sensitivity, as measured by the Matsuda index, plays a crucial role in determining glucose metabolism, both with and without considering the effect of LEO. However, the loss of significance in the GLM analysis could indicate other factors might influence FG more substantially. The potential importance of circadian regulation remains relevant even though the time of LEO was fixed for all participants. This could be due to inherent individual differences in circadian rhythms and how the body processes glucose throughout the night [38,39]. For instance, participants who are naturally evening/night chronotypes (e.g., night owls) may exhibit a different metabolic response to food consumed in the evening compared to early risers, influenced by the timing of their physiological processes—how an individual’s internal clock aligns with external cues [40]. This response may also be regulated by the dawn phenomenon, characterized by a rise in glucose levels during early morning hours as a part of the circadian cycle. This phenomenon, which affects 49% of individuals with type 2 diabetes and 36% with prediabetes [41], can lead to elevated FG levels, and it is possible that the nocturnal glucose patterns we observed were influenced by these circadian-driven mechanisms.

These results imply that dietary recommendations should not be limited to the amount of carbohydrate consumed, but also to the time and macronutrient content of the evening meal to help control overnight glucose levels. For people with prediabetes or type 2 diabetes especially, personalized meal timing and composition strategies could lead to better morning glucose control or lower the risk of complications. Furthermore, insulin sensitivity and circadian phenotype may also play a role in the control of nocturnal glucose metabolism highlighting the need for personalized nutrition interventions.

Our study has many strengths. The study design allows us to capitalize on CGM data collected under both free-living and highly controlled conditions, including controlling meal composition and timing. The use of COF and BOF isolated the specific effects of two novel definitions of overnight glucose on next-day FG. The inclusion of a high-risk sample of individuals with overweight or obesity and prediabetes and type 2 diabetes, including all participants studied at baseline in the parent study (NY-TREAT), highlights the power of analysis of glucose excursions in these individuals, opening the door to possible targeted management to control fasting glucose. However, several limitations need to be acknowledged. The relatively short duration and the well-controlled conditions of the one-day intervention may not capture longer-term glucose dynamics under free-living conditions. Due to the small proportion of male participants in both the initial full cohort (n = 9) and COF/BOF subcohort (n = 4), sex-stratified analyses were not conducted. Future studies with more balanced sex distributions are needed to explore potential sex-specific effects. Although metformin may influence insulin sensitivity, we did not exclude these participants (n = 4) due to the small sample number and because its use is common in individuals with early metabolic disturbance, enhancing the external validity of our findings. The inclusion of individuals with both prediabetes and type 2 diabetes may introduce heterogeneity in insulin sensitivity and secretion patterns, reflecting different stages of disease progression. While many FG values observed fell within the normal range (from min 65.3 to max 118.3 mg/dL), this aligns with early dysglycemia in at-risk populations and highlights the diversity of glycemic profiles. As suggested by Ahlqvist et al. in 2018 [42], some individuals may have predominant insulin secretory defects rather than insulin resistance, which may explain near-normal FG levels despite impaired postprandial responses. Chronotype data were not available, which inhibits the ability to fully understand individual differences in circadian rhythms and their influence on overnight glucose metabolism and FG levels. Moreover, CGM-derived postprandial responses are subject to considerable intra-individual variability, as highlighted in recent studies showing weak reproducibility of glucose excursions even to identical meals [43,44]. Further studies should be performed on larger, more diverse populations with an extended period of monitoring, with systemic assessment of the effect of varying the timing of the LEO and the carbohydrate content range to see whether these findings persist for several days or weeks.

## 5. Conclusions

In conclusion, our results indicate that while the carbohydrate content of the last evening meal does not positively correlate with next-morning glucose levels, the postprandial glucose excursion plays a significant role in overnight glucose levels and, consequently, on fasting glucose levels. The interplay between overnight glucose and insulin sensitivity suggests that both the carbohydrate content of last evening meal and insulin sensitivity may interact in complex ways. This highlights the need for further investigation to elucidate the underlying mechanisms driving nocturnal glucose regulation, including sleep and other biomarkers of circadian rhythms, and consider their potential implications for long-term metabolic health in individuals with overweight or obesity and prediabetes or type 2 diabetes.

## Figures and Tables

**Table 1:** Correlation between Last Eating Occasion (LEO)–Postprandial Glucose Response (PPGR) glucose variables and next-day fasting glucose (FG) (n = 33).

		Next-Day FG	
		r	*p* Value
LEO-PPGR	Mean Glucose (mg/dL)	0.704	<0.001
	Glucose Peak (mg/dL)	0.535	0.001
	Glucose tAUC (min. mg/dL)	0.708	<0.001

## Data Availability

The datasets generated during and/or analyzed during the current study are not publicly available due to being part of an active parent grant with not already published results but are available from the corresponding author upon reasonable request.

## Supplementary Materials

The following supporting information can be downloaded at: https://www.mdpi.com/article/10.3390/nu17122026/s1, Supplementary Table S1. Nutritional composition of the Last Eating Occasion (LEO) across participants (n = 33); Supplementary Table S2. Correlation between LEO carbohydrate content and glucose variables during LEO-PPGR; and association of glucose variables during LEO-PPGR on next-day fasting glucose (FG) adjusted for carbohydrate content (grams and percentage) (n = 33); Supplementary Table S3. Baseline characteristics of the initial cohort (n = 33), final analytical sample for COF and BOF (n = 19) and excluded cases for COF and BOF subcohort (n = 14). Supplementary Table S4. Correlation between COF, BOF, mean glucose average, and carbohydrate content, and association of COF and BOF mean glucose on next-day fasting glucose (FG) adjusted for carbohydrate content (grams and percentage) (n = 19); Supplementary Table S5. Correlation between LEO sugar and fiber content and glucose variables during LEO-PPGR, COF and BOF; and association of LEO-PPGR, COF and BOF glucose variables on next-day fasting glucose (FG) adjusted by sugar or dietary fiber (n = 33 for LEO-PPGR and n = 19 for COF and BOF); Supplementary Table S6. Association of LEO-PPGR, COF and BOF glucose variables on next-day Fasting Glucose (FG) adjusted by Matsuda index (n = 33 for LEO-PPGR and n = 19 for COF and BOF).

## Abbreviations

The following abbreviations are used in this manuscript:

BOF	Biological Overnight Fast
CGM	Continuous Glucose Monitor
COF	Chronological Overnight Fast
CRC	Clinical Research Center
FG	Next-day Fasting Glucose
HbA1c	Hemoglobin A1c
LEO	Last Eating Occasion of the day
LEO-PPGR	The 3h Postprandial Glucose Response after Last Eating Occasion
mCC	myCircadianClock
NY-TREAT	New York Time-Restricted EATing
OGTT	Oral Glucose Tolerance Test
PPGR	Postprandial Glucose Response
tAUC	Total Area Under the Curve

## References

[1] ElSayed N.A., Aleppo G., Bannuru R.R., Bruemmer D., Collins B.S., Ekhlaspour L., Gaglia J.L., Hilliard M.E., Johnson E.L., American Diabetes Association Professional Practice Committee (2023). 2. Diagnosis and Classification of Diabetes: Standards of Care in Diabetes—2024. Diabetes Care.

[2] Gannon M.C., Nuttall F.Q., Westphal S.A., Fang S., Ercan-Fang N. (1998). Acute metabolic response to high-carbohydrate, high-starch meals compared with moderate-carbohydrate, low-starch meals in subjects with type 2 diabetes. Diabetes Care.

[3] Arauz-Pacheco C., Clements G., Cercone S., Brinkley L., Raskin P. (1998). Effects of a large supper on glucose levels the following morning in patients with type 2 diabetes. J. Diabetes Its Complicat..

[4] Mason I.C., Qian J., Adler G.K., Scheer F.A.J.L. (2020). Impact of circadian disruption on glucose metabolism: Implications for type 2 diabetes. Diabetologia.

[5] Basu R., Barosa C., Jones J., Dube S., Carter R., Basu A., Rizza R.A. (2013). Pathogenesis of Prediabetes: Role of the Liver in Isolated Fasting Hyperglycemia and Combined Fasting and Postprandial Hyperglycemia. J. Clin. Endocrinol. Metab..

[6] Porter J., Horne J. (1981). Bed-time food supplements and sleep: Effects of different carbohydrate levels. Electroencephalogr. Clin. Neurophysiol..

[7] Rizza R.A. (2010). Pathogenesis of fasting and postprandial hyperglycemia in type 2 diabetes: Implications for therapy. Diabetes.

[8] Phillip M., Battelino T., Atlas E., Kordonouri O., Bratina N., Miller S., Biester T., Stefanija M.A., Muller I., Nimri R. (2013). Nocturnal glucose control with an artificial pancreas at a diabetes camp. N. Engl. J. Med..

[9] Ceriello A., Monnier L., Owens D. (2019). Glycaemic variability in diabetes: Clinical and therapeutic implications. Lancet Diabetes Endocrinol..

[10] Liarakos A.L., Lim J.Z.M., Leelarathna L., Wilmot E.G. (2024). The use of technology in type 2 diabetes and prediabetes: A narrative review. Diabetologia.

[11] Hall H., Perelman D., Breschi A., Limcaoco P., Kellogg R., McLaughlin T., Snyder M., Locasale J. (2018). Glucotypes reveal new patterns of glucose dysregulation. PLOS Biol..

[12] Lejk A., Chrzanowski J., Cieślak A., Fendler W., Myśliwiec M. (2022). Reduced Carbohydrate Diet Influence on Postprandial Glycemia—Results of a Short, CGM-Based, Interventional Study in Adolescents with Type 1 Diabetes. Nutrients.

[13] Devlin B.L., Parr E.B., Radford B.E., Hawley J.A. (2020). Lower nocturnal blood glucose response to a potato-based mixed evening meal compared to rice in individuals with type 2 diabetes. Clin. Nutr..

[14] Gill S., Panda S. (2015). A Smartphone App Reveals Erratic Diurnal Eating Patterns in Humans that Can Be Modulated for Health Benefits. Cell Metab..

[15] Santos–Báez L.S., Garbarini A., Shaw D., Cheng B., Popp C.J., Manoogian E.N., Panda S., Laferrère B. (2022). Time-restricted eating to improve cardiometabolic health: The New York Time-Restricted EATing randomized clinical trial—Protocol overview. Contemp. Clin. Trials.

[16] Manoogian E.N.C., Wei-Shatzel J., Panda S. (2022). Assessing temporal eating pattern in free living humans through the myCircadianClock app. Int. J. Obes..

[17] Karlsson M., Olsson E., Becker W., Karlström B., Cederholm T., Sjögren P. (2017). Ability to predict resting energy expenditure with six equations compared to indirect calorimetry in octogenarian men. Exp. Gerontol..

[18] Mifflin M.D., St Jeor S.T., Hill L.A., Scott B.J., Daugherty S.A., Koh Y.O. (1990). A new predictive equation for resting energy expenditure in healthy individuals. Am. J. Clin. Nutr..

[19] Gutch M., Kumar S., Razi S.M., Gupta K.K., Gupta A. (2015). Assessment of insulin sensitivity/resistance. Indian J. Endocrinol. Metab..

[20] de Almeida R.S., Marot L.P., Latorraca C.d.O.C., Oliveira R.d.Á., Crispim C.A. (2022). Is Evening Carbohydrate Intake in Healthy Individuals Associated with Higher Postprandial Glycemia and Insulinemia When Compared to Morning Intake? A Systematic Review and Meta-Analysis of Randomized Crossover Studies. J. Am. Nutr. Assoc..

[21] Nakamura K., Tajiri E., Hatamoto Y., Ando T., Shimoda S., Yoshimura E. (2021). Eating Dinner Early Improves 24-h Blood Glucose Levels and Boosts Lipid Metabolism after Breakfast the Next Day: A Randomized Cross-Over Trial. Nutrients.

[22] Díaz-Rizzolo D.A., Baez L.S.S., Popp C.J., Borhan R., Sordi-Guth A., Manoogian E.N.C., Panda S., Cheng B., Laferrère B. (2024). Late eating is associated with poor glucose tolerance, independent of body weight, fat mass, energy intake and diet composition in prediabetes or early onset type 2 diabetes. Nutr. Diabetes.

[23] Shrestha L., Jha B., Yadav B., Sharma S. (2013). Correlation between fasting blood glucose, postprandial blood glucose and glycated hemoglobin in non-insulin treated type 2 diabetic subjects. Sunsari Tech. Coll. J..

[24] Monnier L., Lapinski H., Colette C. (2003). Contributions of fasting and postprandial plasma glucose increments to the overall diurnal hyperglycemia of type 2 diabetic patients: Variations with increasing levels of HbA(1c). Diabetes Care.

[25] Ben-Yacov O., Godneva A., Rein M., Shilo S., Kolobkov D., Koren N., Dolev N.C., Shmul T.T., Wolf B.C., Kosower N. (2021). Personalized Postprandial Glucose Response–Targeting Diet Versus Mediterranean Diet for Glycemic Control in Prediabetes. Diabetes Care.

[26] Jarrett R.J., Keen H. (1969). Diurnal variation of oral glucose tolerance: A possible pointer to the evolution of diabetes mellitus. BMJ.

[27] Van Cauter E., Polonsky K.S., Scheen A.J. (1997). Roles of circadian rhythmicity and sleep in human glucose regulation. Endocr. Rev..

[28] Nuttall F.Q., Gannon M.C. (2004). Metabolic response of people with type 2 diabetes to a high protein diet. Nutr. Metab..

[29] Axelsen M., Lönnroth P., Lenner R.A., Taskinen M.-R., Smith U. (2000). Suppression of nocturnal fatty acid concentrations by bedtime carbohydrate supplement in type 2 diabetes: Effects on insulin sensitivity, lipids, and glycemic control. Am. J. Clin. Nutr..

[30] Dyer-Parziale M. (2001). The effect of extend bar containing uncooked cornstarch on night-time glycemic excursion in subjects with type 2 diabetes. Diabetes Res. Clin. Pract..

[31] Axelsen M., Lenner R.A., Lönnroth P., Smith U. (1999). Breakfast glycaemic response in patients with type 2 diabetes: Effects of bedtime dietary carbohydrates. Eur. J. Clin. Nutr..

[32] Axelsen M., Lönnroth P., Lenner R.A., Smith U. (2003). Suppression of the nocturnal free fatty acid levels by bedtime cornstarch in NIDDM subjects. Eur. J. Clin. Investig..

[33] Nuttall F.Q., Almokayyad R.M., Gannon M.C. (2015). Comparison of a carbohydrate-free diet vs. fasting on plasma glucose, insulin and glucagon in type 2 diabetes. Metabolism.

[34] Fromentin C., Tomé D., Nau F., Flet L., Luengo C., Azzout-Marniche D., Sanders P., Fromentin G., Gaudichon C. (2013). Dietary proteins contribute little to glucose production, even under optimal gluconeogenic conditions in healthy humans. Diabetes.

[35] Abbie E., Francois M.E., Chang C.R., Barry J.C., Little J.P. (2020). A low-carbohydrate protein-rich bedtime snack to control fasting and nocturnal glucose in type 2 diabetes: A randomized trial. Clin. Nutr..

[36] Boden G., Chen X., Urbain J.L. (1996). Evidence for a circadian rhythm of insulin sensitivity in patients with NIDDM caused by cyclic changes in hepatic glucose production. Diabetes.

[37] Stumvoll M., Tataranni P.A., Stefan N., Vozarova B., Bogardus C. (2003). Glucose allostasis. Diabetes.

[38] Isherwood C.M., van der Veen D.R., Hassanin H., Skene D.J., Johnston J.D. (2023). Human glucose rhythms and subjective hunger anticipate meal timing. Curr. Biol..

[39] Jones S.E., Lane J.M., Wood A.R., van Hees V.T., Tyrrell J., Beaumont R.N., Jeffries A.R., Dashti H.S., Hillsdon M., Ruth K.S. (2019). Genome-wide association analyses of chronotype in 697,828 individuals provides insights into circadian rhythms. Nat. Commun..

[40] Stutz B., Krueger B., Goletzke J., Jankovic N., Alexy U., Herder C., Dierkes J., Berg-Beckhoff G., Jakobsmeyer R., Reinsberger C. (2024). Glycemic response to meals with a high glycemic index differs between morning and evening: A randomized cross-over controlled trial among students with early or late chronotype. Eur. J. Nutr..

[41] Barua S., Glantz N., Larez A., Bevier W., Sabharwal A., Kerr D. (2024). A probabilistic computation framework to estimate the dawn phenomenon in type 2 diabetes using continuous glucose monitoring. Sci. Rep..

[42] Ahlqvist E., Storm P., Käräjämäki A., Martinell M., Dorkhan M., Carlsson A., Vikman P., Prasad R.B., Aly D.M., Almgren P. (2018). Novel subgroups of adult-onset diabetes and their association with outcomes: A data-driven cluster analysis of six variables. Lancet Diabetes Endocrinol..

[43] Hutchins K.M., Betts J.A., Thompson D., Hengist A., Gonzalez J.T. (2025). Continuous glucose monitor overestimates glycemia, with the magnitude of bias varying by postprandial test and individual—A randomized crossover trial. Am. J. Clin. Nutr..

[44] Hengist A., Ong J.A., McNeel K., Guo J., Hall K.D. (2024). Imprecision nutrition? Intraindividual variability of glucose responses to duplicate presented meals in adults without diabetes. Am. J. Clin. Nutr..

