# Supplementary material for: Biological vs. Chronological Overnight Fasting: Influence of Last Evening Meal on Morning Glucose in Dysglycemia
Source: Nutrients. 2025 Jun 18;17(12):2026. doi: 10.3390/nu17122026 (PMC12195734; doi:10.3390/nu17122026)
Supplement: Supplementary file 1 [file nutrients-17-02026-s001.zip › nutrients-3662786-supplementary.pdf]

Supplementary Table S1. Nutritional composition of the Last Eating Occasion (LEO) across participants (N=33).

	Mean (SD)	Minimum range value	Maximum range value
Energy (kcal)	207.2 (36.0)	163.0	359.0
Carbohydrate (g)	21.4 (14.3)	6.0	56.0
Carbohydrate (%)	40.8 (23.9)	14.3	88.4
Protein (g)	5.7 (1.9)	3.5	12.6
Protein (%)	9.4 (2.1)	6.5	12.2
Fat (g)	12.5 (6.7)	1.3	31.5
Fat (%)	51.5 (22.4)	5.1	73.5
Sugar (g)	7.6 (5.9)	1.4	21.9
Sugar (%)	13.9 (10.8)	0.2	40.4
Dietary Fiber (g)	5.2 (1.5)	2.2	6.8

grams: g, standard deviation: SD.

Supplementary Table S2. Correlation between LEO carbohydrate content and glucose variables during LEO-PPGR; and association of glucose variables during LEO-PPGR on next-day Fasting Glucose (FG) adjusted for carbohydrate content (grams and percentage) (n=33).

		LEO-PPGR					
Methods	Variables	Mean glucose (mg/dL)		Peak glucose (mg/dL)		tAUC (min*mg/dL)	
Pearson		r	p-value	r	p-value	r	p-value
	Carbohydrate (g)	-0.152	0.398	0.163	0.366	-0.157	0.382
	Carbohydrate (%)	-0.078	0.667	0.224	0.211	-0.084	0.641
GLM		B	p-value	B	p-value	B	p-value
	Next-day FG (mg/dL) by carbohydrate (g)	-0.015	0.052	-0.014	0.057	<-0.001	**0.048**
	Next-day FG (mg/dL) by carbohydrate (%)	-0.01	0.057	-0.009	0.056	<-0.001	0.054

Fixed-3h Postprandial Glucose Response after Last Eating Occasion: LEO-PPGR, Fasting Glucose: FG; total Area Under the Curve; tAUC; General Linear Model: GLM; grams: g.

Supplementary Table S3. Baseline characteristics of the initial cohort (n=33), final analytical sample for COF and BOF (n=19) and excluded cases for COF and BOF subcohort (n=14).

	Initial cohort (n=33)	COF/BOF subcohort (n=19)	Excluded cases subcohort (n=14)	p-value *	p-value ¥
Women (%)	72.73	78.95	71.43	0.350	0.201
Type 2 diabetes diagnosis (%)	9.09	10.53	7.14	0.738	0.509
Metformin use (%)	12.12	15.79	14.29	0.905	0.680
Waist circumference (cm)	105.54 (14.53)	103.72 (16.04)	108.19 (12.09)	0.680	0.371
Body weight (kg)	93.02 (22.30)	87.85 (20.79)	100.03 (23.11)	0.414	0.872
BMI (kg/m^2)	33.48 (6.56)	31.95 (5.01)	35.54 (7.95)	0.386	0.384
HbA1c (%)	5.97 (0.23)	5.95 (0.24)	5.99 (0.22)	0.799	0.902

Percentage (%) or mean in units (standard deviation: SD). Categorical variables were compared using the Chi-square test, and continuous variables using the Student's t-test.

* Initial cohort vs COF/BOF subcohort

¥ COF/BOF subcohort vs excluded cases subcohort

Supplementary Table S4. Correlation between Chronological Overnight Fast (COF) Biological Overnight Fast (BOF) mean glucose average and carbohydrate content; and association of COF and BOF mean glucose on next-day Fasting Glucose (FG) adjusted for carbohydrate content (grams and percentage) (n=19).

Method	Variable	COF mean glucose (mg/dL)		BOF mean glucose (mg/dL)	
Pearson		r	p-value	r	p-value
	Next-day FG (mg/dL)	0.878	**<0.001**	0.878	**<0.001**
	Carbohydrate (g)	-0.309	0.199	-0.437	0.061
	Carbohydrate (%)	-0.246	0.310	-0.369	0.120
GLM		B	p-value	B	p-value
	Next-day FG (mg/dL) by carbohydrate (g)	-0.016	0.106	-0.013	0.191
	Next-day FG (mg/dL) by carbohydrate (%)	-0.012	0.061	-0.010	0.127

Fasting Glucose: FG; Chronological Overnight Fast: COF, Biological Overnight Fast: BOF, General Linear Model: GLM; grams: g.

Supplementary Table S5. Correlation between LEO sugar and fiber content and glucose variables during LEO-PPGR, COF and BOF; and association of LEO-PPGR, COF and BOF glucose variables on next-day Fasting Glucose (FG) adjusted by sugar or dietary fiber (n=33 for LEO-PPGR and n=19 for COF and BOF).

		LEO-PPGR						COF		BOF	
Method	Variable	Mean glucose (mg/dL)		Peak glucose (mg/dL)		tAUC (min*mg/dL)		Mean glucose (mg/dL)		Mean glucose (mg/dL)	
Pearson		r	p-value	r	p-value	r	p-value	r	p-value	r	p-value
	Sugar (g)	0.079	0.643	0.266	0.111	0.080	0.638	-0.224	0.357	-0.346	0.147
	Sugar (%)	0.141	0.404	0.314	*0.059*	0.142	0.402	-0.143	0.558	-0.256	0.291
	Dietary fiber (g)	0.428	**0.008**	0.350	**0.034**	0.424	**0.009**	0.218	0.370	0.359	0.131
GLM		B	p-value	B	p-value	B	p-value	B	p-value	B	p-value
	Next-day FG (mg/dL) by sugar (g)	-0.037	0.063	-0.034	0.092	<-0.001	0.059	-0.046	0.131	-0.039	0.189
	Next-day FG (mg/dL) by sugar (%)	-0.023	0.083	-0.020	0.134	<-0.001	0.079	-0.032	0.099	-0.027	0.141
	Next-day FG (mg/dL) by dietary fiber (g)	0.085	0.286	0.159	0.041	<-0.001	0.282	0.115	0.144	0.079	0.332

Fixed-3h Postprandial Glucose Response after Last Eating Occasion: LEO-PPGR; Fasting Glucose: FG, total Area Under the Curve; tAUC, Chronological Overnight Fast: COF, Biological Overnight Fast: BOF, General Linear Model: GLM; grams: g.

Supplementary Table S6. Association of LEO-PPGR, COF and BOF glucose variables on next-day Fasting Glucose (FG) adjusted by Matsuda index (n=33 for LEO-PPGR and n=19 for COF and BOF).

		LEO-PPGR						COF		BOF	
Method	Variable	Mean glucose (mg/dL)		Peak glucose (mg/dL)		tAUC (min*mg/dL)		Mean glucose (mg/dL)		Mean glucose (mg/dL)	
		B	p-value	B	p-value	B	p-value	B	p-value	B	p-value
GLM	Next-day FG (mg/dL) by Matsuda index	-0.021	0.711	-0.039	0.548	<-0.001	0.713	-0.081	0.169	-0.062	0.239

Fixed-3h Postprandial Glucose Response after Last Eating Occasion: LEO-PPGR; Fasting Glucose: FG, total Area Under the Curve; tAUC, Chronological Overnight Fast: COF, Biological Overnight Fast: BOF, General Linear Model: GLM; grams: g.
